# Contactless Camera-Based Heart Rate and Respiratory Rate Monitoring Using AI on Hardware

**DOI:** 10.3390/s23094550

**Published:** 2023-05-07

**Authors:** Dimitrios Kolosov, Vasilios Kelefouras, Pandelis Kourtessis, Iosif Mporas

**Affiliations:** 1 School of Physics, Engineering and Computer Science, University of Hertfordshire, Hatfield AL10 9AB, UK; p.kourtessis@herts.ac.uk; 2 School of Engineering, Computing and Mathematics, University of Plymouth, Plymouth PL4 8AA, UK; vasilios.kelefouras@plymouth.ac.uk

**Keywords:** embedded systems, AI/ML health monitoring algorithms, efficient health monitoring hardware platforms, real-time health monitoring

## Abstract

Detecting vital signs by using a contactless camera-based approach can provide several advantages over traditional clinical methods, such as lower financial costs, reduced visit times, increased comfort, and enhanced safety for healthcare professionals. Specifically, Eulerian Video Magnification (EVM) or Remote Photoplethysmography (rPPG) methods can be utilised to remotely estimate heart rate and respiratory rate biomarkers. In this paper two contactless camera-based health monitoring architectures are developed using EVM and rPPG, respectively; to this end, two different CNNs, (Mediapipe’s BlazeFace and FaceMesh) are used to extract suitable regions of interest from incoming video frames. These two methods are implemented and deployed on four off-the-shelf edge devices as well as on a PC and evaluated in terms of latency (in each stage of the application’s pipeline), throughput (FPS), power consumption (Watt), efficiency (throughput/Watt), and value (throughput/cost). This work provides important insights about the computational costs and bottlenecks of each method on each hardware platform, as well as which platform to use depending on the target metric. One of our insights shows that the Jetson Xavier NX platform is the best platform in terms of throughput and efficiency, while Raspberry Pi 4 8 GB is the best platform in terms of value.

## 1. Introduction

Heart rate and respiratory rate are crucial biomarkers whose anomalous patterns can indicate various health conditions. Detecting such biomarkers using contactless camera-based health monitoring methods provides several advantages over traditional clinical methods, such as lower financial costs, reduced visit times, increased comfort, and enhanced safety for healthcare professionals [1]. Eulerian Video Magnification (EVM) and remote Photoplethysmography (rPPG) typify such contactless camera-based health monitoring methods to estimate human vital signs, e.g., heart rate (HR) and respiratory rate (RR).

Developing efficient contactless camera-based health monitoring applications is a non-trivial and challenging task for several reasons. First, the input video normally suffers from low SNR and high variability in PPG estimation due to sensor–subject angles, different types of cameras, or exposed light types [1]. Second, these applications are normally both compute- and memory intensive and therefore their deployment in resource-limited edge devices is not always feasible. Third, a wide range of computer vision and signal processing models and techniques are available, providing different trade-offs between accuracy and complexity. Fourth, a wide range of edge devices exist, with diverse hardware architectures, providing trade-offs among throughput (processed Frames per Second (FPS)), development time, energy consumption, financial cost, efficiency (throughput/watt), and value (throughput/cost).

In this article, we present two contactless camera-based health monitoring architectures that can estimate the vital signs (heart rate and respiratory rate) of an individual from distance. To this end, the EVM [2,3,4,5] and rPPG [4,6] widely used methods are implemented and deployed on four off-the-shelf edge devices as well as on a PC; these edge devices are: (a) Raspberry Pi 4 4 GB with 32-bit OS (RP4_32bit), (b) Raspberry Pi 4 8 GB with 64-bit OS (RP4_64bit), (c) Jetson Nano, (d) Jetson Xavier NX. The regions of interest (ROIs) are extracted from each frame by using computer vision and in particular two different Convolutional Neural Networks (CNNs). 

A thorough performance evaluation of the entire end-to-end application (full video pipeline) is performed, including all application steps (e.g., pre/post processing, reading the input frame) and various performance metrics. Furthermore, the five hardware platforms are compared in terms of throughput (FPS), value (throughput/cost), and efficiency (throughput/Watt). We provide important insights around the capabilities and bottlenecks of each hardware platform as well as which platform to use depending on the target metric. We show that Jetson Xavier NX is the best platform in terms of throughput and efficiency; meanwhile, Raspberry Pi 4 8 GB is the best platform in terms of value. Last, we show that rPPG achieves a higher throughput compared to EVM.

This research work has resulted in the following contributions:

- The development of two contactless camera-based health monitoring architectures for edge devices, estimating heart rate and respiratory rate.
- The evaluation and comparison of five hardware platforms in terms of throughput (FPS), value (throughput/cost), and efficiency (throughput/Watt) metrics, when running camera-based health monitoring software applications.
- Important insights regarding the capabilities of each hardware platform, which can inform the selection of a platform based on the target metric or metrics.
- An overview of the computational cost of each application stage, with identified bottlenecks.

The remainder of this paper is organized as follows. Section 2 reviews the related work and Section 3 describes the system architecture of the edge devices. In Section 4, the experimental setup is presented. In Section 5, the experimental results are shown and discussed and finally, Section 6 is dedicated to conclusions.

## 2. Related Work

The estimation of biomarkers can be separated into two categories, contact-based and contactless. Various medical devices today utilise contact-based approaches to determine physiological measurements around a person’s health condition. A popular contact-based approach is electrocardiography (ECG) [7] where by attaching electrode sensors to a person’s skin, the observed voltage between heart beats can be used to derive the heart’s rhythm. Another popular method is photoplethysmography (PPG) [8,9], which takes a different approach and measures the volumetric changes in blood with infrared light placed upon the skin. While ECG is more accurate, PPG is a less intrusive and lower cost solution, and therefore it is normally used as a reference point for evaluation of various PPG methods [10]. Although contact-based methods are more accurate, contactless methods are popular too, since they provide several advantages such as lower financial costs, reduced visit times, and increased comfort. Two popular contactless methods are Eulerian Video Magnification (EVM) and remote photoplethysmography (rPPG), which are the topic of this paper. 

The remainder of the related work section is structured into three subsections. Section 2.1 discusses EVM, Section 2.2 covers rPPG, and Section 2.3 outlines the various health monitoring implementations on edge hardware. Each subsection provides a detailed discussion of the topic, including relevant research and findings.

### 2.1. Eulerian Video Magnification (EVM)

Eulerian video magnification (EVM) has been shown to be highly effective for non-contact, unobtrusive, and non-invasive patient heart-rate estimation systems [11]. The EVM approach was developed at the Computer Science and Artificial Intelligence Lab of the Massachusetts Institute of Technology (MIT CSAIL) in 2012 [12] and can be used to enhance motion or colour in order to reveal information hidden to the naked eye. By applying this to multiple video frames, enhancing the colours, and plotting the colour variations over time, one can process that and derive the heart rate and other vital signs without making physical contact with the patient.

Regarding the ability of EVM to estimate heart rate and respiratory rate, it is important to note that there is a lack of common datasets; however, various previous research studies have evaluated EVM by using their own datasets and different metrics, and their reported performance has shown the validity of EVM in this task. Previous studies have reported accuracies of 94.0% from eleven subjects [2] and 93.0% from one subject [5], based on Equation (1). In [4], evaluations with twenty subjects were performed, and the performance was evaluated in terms of absolute errors, resulting in 98.0% and 96.6% accuracy based on Equation (2) for supervised and unsupervised EVM approaches, respectively. In [13], the authors evaluated EVM on RGB video streams using two human subjects and two monkey subjects, measuring the mean pulse rates with an accuracy equal to 93.2% for humans and 97.3% for monkeys based on Equation (1). For respiratory rate estimation via EVM, an error rate of 1.5% was reported, which based on Equation (1) equals 98.5% accuracy [14].

(1) $$Accuracy=100-100\times \left(\frac{\left|Estimation-Real\right|}{Estimation}\right)$$

(2) $$Accuracy=100-\left|error\right|$$

Since the introduction of EVM, various efforts have been made to further investigate and improve the robustness of this approach. For example, the effectiveness can be improved by using temporal [15] or spatio-temporal [16] filtering, while other efforts focused on feeding specific region of interests [17] in order to remove redundant data from the image frame.

### 2.2. Remote Photoplethysmography (rPPG)

Similar to photoplethysmography (PPG), remote photoplethysmography (rPPG) is another approach for the contactless estimation of the heart rate and other vital signs; rPPG detects blood volume changes by capturing pixel intensity changes from the skin [18]. During cardiac cycles, changes in blood volume cause changes on the skin, which can be picked up by optical sensors [19] and a plethysmography signal can be derived and used to estimate biomarkers. There have been variations in the algorithm (in terms of the method) used to extract and process the data, with Table 1 showing some of the most popular signal extraction methods. In [20], eight image-based photoplethysmography (iPPG) extraction methods (GRD, AGRD, PCA, ICA, LE, SPE, CHROM, and POS) were compared in terms of Spearman correlation and Normalized Root-Mean-Square Error (NRMSE).

Previous studies used different datasets and metrics to evaluate the accuracy of rPPG method for heart rate estimation. A study involving 140 subjects reported an error (mean difference of estimated and ground truth) of 2.0% [18]. Other studies evaluated rPPG heart rate estimation using various metrics such as MAE (Mean Absolute Error), r (Pearson correlation coefficient), SNR (Signal-to-Noise Ratio), and TMC (Template Match Correlation) with the reported MAE on the VIPL dataset of 3.9 bpm [30], and r and MAE values of 0.86 and 3.14%, respectively, for 67 subjects with underlying cardiovascular disease [31]. For respiratory rate estimation, RMSE results of 1.7014 and 2.5026 were reported using Hue (HSV colour space) and GREEN rPPG methods, respectively [32]. Additionally, a method was developed in [33] to detect influenza using rPPG to estimate HR and RR, with a reported r of 0.87 for both. 

### 2.3. Health Monitoring on Edge Hardware

Developing and deploying efficient contactless camera-based health monitoring applications on resource-limited edge devices introduces several challenges as the computation and memory requirements of these applications are normally high. The most popular techniques to address this problem are briefly reported below. 

First, lightweight computer vision models have been developed, that adopt various innovative techniques to reduce the number of parameters and tensor operations while maintaining satisfactory accuracy [34]; a wide range of computer vision and signal processing models are available, providing different trade-offs between accuracy and complexity. For our use case, artificial intelligence (AI) models serve the purpose of identifying the regions of interest (ROIs) within a given frame, with the objective of providing the most appropriate data to facilitate algorithmic estimation of heart rate and respiratory rate. Consequently, in the context of developing systems targeted for the edge, it is imperative to comprehend the trade-offs between the quality of the models, computational costs, commercial costs, and power consumption. In [35,36], several computer vision models are evaluated and compared to six popular edge devices in terms of accuracy and inference time.

Second, various hardware platforms (accelerators) have been designed with diverse hardware architectures, such as NVIDIA Jetson, Intel NCS2, Google Edge TPU, and others, providing trade-offs between latency time, development time, energy consumption, financial cost, efficiency (throughput/watt), and value (throughput/cost). Such hardware platforms offer various benefits such as energy efficiency, ultra-low latency, and low financial costs, that allow the efficient deployment of health applications on the edge; something that was not feasible previously [37]. 

There have been various research studies covering contactless biomarker estimation; however, the majority of them do not employ a common dataset for validation and they focus only on a few hardware performance metrics. In [37], a contactless rPPG pulse-rate detection system with face recognition was developed on an Nvidia Jetson TX2 platform, but only explored accuracy results on their custom dataset. In [38], an rPPG solution was developed on an Nvidia Jetson AGX Xavier too, exploring only MAE and FPS metrics. Field Programmable Gate Arrays (FPGAs) have also been used in this area and are an excellent choice for custom health application implementations because of their power efficiency, latency, throughput, flexibility in interfaces, and reconfigurability; in [39], an rPPG implementation was proposed where the heart rate estimation processing part runs on a soft CPU while the rest of the system is implemented on the reconfigurable logic. Note that to leverage such complex hardware platforms, advanced optimization frameworks are needed, such as TFLITE [40] for ARM microcontrollers and microprocessors and TensorRT for Nvidia edge GPUs [41].

Furthermore, compression-based techniques have been introduced, such as quantization [34], weight pruning [42], and low rank factorization [43]; these techniques reduce both the memory size of the model and the number of executed instructions, by normally sacrificing accuracy. In [1], quantization and pruning were used when detecting changes in blood volume on three edge devices.

Compared to all the aforementioned methods, this work evaluates two end-to-end contactless camera-based health monitoring applications, first, on a wide range of off-the-shelf edge devices and second, by using several metrics such throughput, energy consumption, value (throughput/cost), and efficiency (throughput/Watt).

## 3. System Architecture

The system architecture was implemented with the intention of deploying EVM and rPPG on various edge devices, which would have limited computational capabilities in terms of memory and processing speed. For both approaches, the estimation of heart rate and respiratory rate relies on buffering data, specifically regions of interest (ROIs) obtained through CNNs, prior to executing the corresponding algorithms.

This section is divided into three subsections. Section 3.1 describes the general block diagram of the system architecture aimed for the hardware platforms. This is followed by Section 3.2 and Section 3.3 providing a more detailed explanation of the implementations steps of EVM and rPPG, respectively.

### 3.1. Edge Device System Architecture

**Figure 1:**
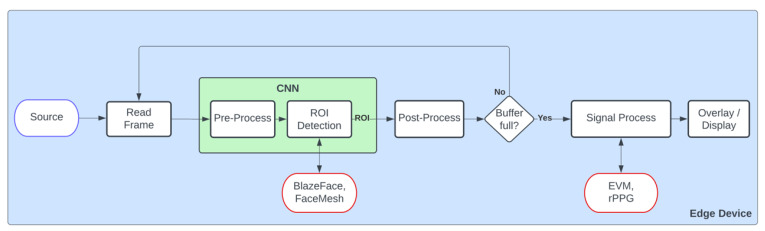
Generic system block diagram of edge devices.

In this subsection, we present the general system architecture, which is designed for edge device deployment (see Figure 1). The flow of the system is similar for both the EVM and rPPG approaches, and each step of the data pipeline is explained in further detail below.

1. Source

The source of data input can be a video file or live video streaming from a USB webcam. For the benchmarking performance of each edge device, a recorded video file of 11 s long was used in three different resolutions, 1920 × 1080 (1080p), 1280 × 720 (720p) and 640 × 360 (360p), at 30 frames per second.

2. Read Frame

Reading a frame from either a webcam or a video file would have different processing times depending on the resolution and processing capabilities of the CPU. For EVM, it possesses an additional function of resizing the frame to 640 × 360, as anything bigger than that will cause most of the edge devices to run out of memory and crash the application, while for rPPG the target frame size is maintained.

3. CNN

The CNN stage has two parts, with a pre-process step being the first. Its purpose is to format the incoming frame to be compatible with the target CNN requirements, e.g., change the input resolution. For ROI detection, two different CNN models were used, both from Google’s MediaPipe tool [44]. The first one, a face detection model based on BlazeFace [45], is an ultrafast face-detection solution that besides estimating bounding boxes, also displays six face landmarks (not used in this instance). It accepts 128 × 128 input resolution, and it is based on MobileNetV1/V2 architecture, with three very distinct differences. The first difference is that it uses 5 × 5 kernel sizes for its depthwise separable convolutions, as it was found that increasing the kernel size is relative cheap. The second difference is that it uses a modified version of the popular Single Shot Detection (SSD) method [46], aimed at more effective mobile GPU utilization. Third, it uses a blending strategy, an alternative post-processing algorithm to non-maximum suppression (NMS) which the authors stated provided a 10% increase in the accuracy of their results.

The second model, FaceMesh [47] is a 2-step model that estimates 468 3D face landmarks; it accepts 192 × 192 input resolution. It consists of a face detection model (can be any lightweight architecture, but BlazeFace is used) and a face landmark model. The cropped image of the face and several core landmarks are provided by the face detector and then processed by the mesh neural network to produce a vector of 3D landmark coordinates. These coordinates are used to detect and crop the ROIs for our use case, which are the forehead and left/right cheek regions of the detected face, in order to eliminate any redundant areas of the face that do not contribute to HR or RR estimation.

**Figure 2:**
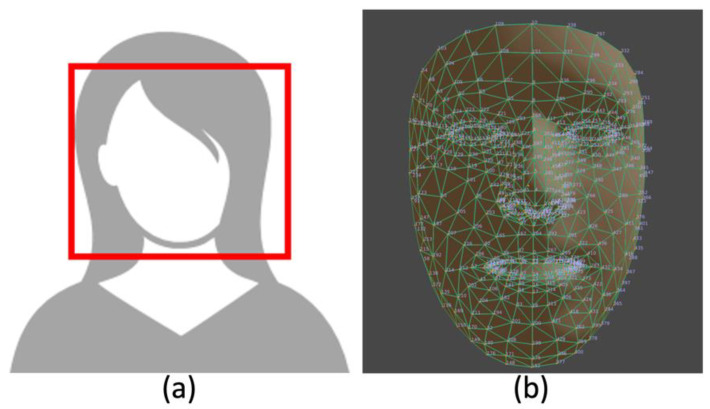
(**a**) BlazeFace (face detection) example [48]. (**b**) FaceMesh (face landmarks) example [49].

Both CNNs (example in Figure 2) were deployed on each platform in their original datatype form (TFLITE FP16 models) with no further optimizations for the target hardware. Optimising each model for the respective target hardware to maximise performance could be a potential case study for future work.

4. Post-Process

In the post-process stage, the CNN model coordinates’ results are processed, and the corresponding ROI is derived from the original frame. For EVM, the ROI are resized to a 180 × 180 resolution due to requiring a squared input (width and length being equal) and a fixed image size for the Signal Processing stage. For rPPG, the green channel of the RGB frame is extracted and then the mean value is calculated.

5. Buffer

Before feeding data for HR/RR estimation (EVM or rPPG), ROIs data are buffered until a sufficient amount is obtained for the signal process stage. In our use case, we used 180 frames for data buffering, until vital signs can start being estimated. After the buffer is full, it acts as a shift register.

6. Signal Process

In the signal process stage, the incoming data are processed by the corresponding algorithm to estimate the heart rate and respiratory rate using either EVM (described in Section 3.2) or rPPG algorithm (described in Section 3.3).

7. Overlay/Display

Lastly, the overlay/display stage is where the processed frame is displayed, showcasing heart rate, respiratory rate, FPS and any overlays, which include pre-processed video data derived from either rPPG or EVM. Performance may vary based on resolution, and the number of overlays.

### 3.2. Eulerian Video Magnification (EVM) Implementation

**Figure 3:**
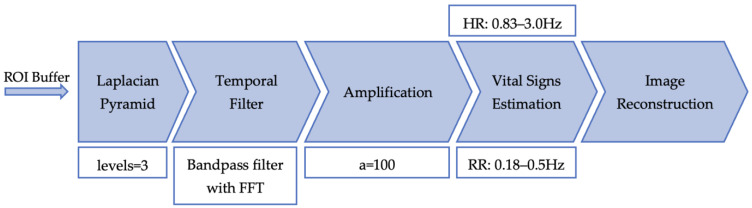
EVM method block diagram.

The EVM method works by decomposing the video frames into distinct spatial frequency bands using full Laplacian pyramids [12]. The Laplacian pyramid is based on the Gaussian pyramid with *l* levels for each frame, which is basically down sampling by a factor of 2 for each level of the pyramid. Then, the spatial image derived from the Laplacian pyramid is converted to the frequency domain via Fast Fourier Transform (FFT) and using a temporal filter the frequency bands of interested are isolated and extracted. In the next stage, the filtered bandpass signal can be amplified by a magnification factor (*a* factor). Then, finding the peaks within those certain frequency bands results in the estimation of vital signs. For heart rate, frequencies of interest are between 0.83 and 3.0 Hz (50 to 180 beats per minute); meanwhile, for respiratory rate, the frequencies of interest are between 0.18 and 0.5 Hz (11 to 30 breaths per minute) [50]. Finally, to reconstruct amplified frames, iteratively each processed frame is up sampled using a Gaussian filter until the size of the original frame is reached, where the variations in colours can be revealed; this is an optional step to visualise subtle changes in colour. The complete flow of EVM is depicted in Figure 3.

### 3.3. Remote Photoplethysmography (rPPG) Implementation

**Figure 4:**
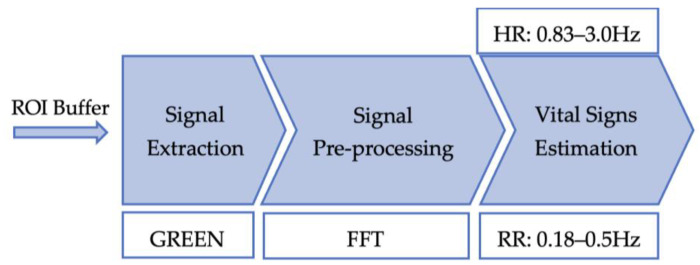
rPPG method block diagram.

The rPPG algorithm can be divided into three key stages; the first stage is the signal extraction of several ROIs frames, the second stage is the signal pre-processing, and the third stage estimates vital signs. The software flowchart used in our implementation approach for rPPG can be seen in Figure 4, depicting the stages that were mentioned.

Given that the target implementation is aimed at resource-constrained edge devices, we selected the least computationally intensive signal extraction method (GREEN in Table 1). In the GREEN method (Table 1), only the green channel is processed and therefore the number of computations is highly reduced; it should be noted that [51] proved that the usage of the green channel results in less signal-to-noise-ratio (SNR) than using all the channel colours of RGB. After extracting and calculating the mean of the green pixel values of multiple ROIs frames, common signal pre-processing techniques are applied to clean and derive the pulse signal. Signal pre-processing starts with detrending, in order to remove unwanted noise from light changes in the frame [30]. Next, by interpolating the detrended signal by one, we obtain an even signal, since its sampling could have been performed at non-periodic intervals. Followed by applying a Hamming window, the signal becomes more periodic and reduces any spectral leakage that might have been introduced. Afterwards, the signal is normalised by dividing it by its L2 norm. Lastly, using a 1D Fast Fourier Transform (FFT), the signal is transformed into the frequency domain. Once in the frequency domain, within the frequency bands of interest, the highest peak of the amplitude spectrum contains the vital signs. Similarly to EVM, the same cut-off frequencies for heart rate (0.83–3.0 Hz) and respiratory rate (0.18–0.5 Hz) were used.

## 4. Experimental Setup

The EVM and rPPG methods were benchmarked on four different commercial off-the-shelf hardware platforms, specifically in terms of their inference times, efficiency, and value. While it may appear meaningless to conduct these evaluations on a PC, given that the objective is to assess the capabilities of edge technology, this was a good reference point for comparison. The embedded hardware setups that were used in this work are shown in Table 2, with their core characteristics described below.

1. PC

Desktop workstation fitted with ×86 CPU (Intel i9-9900K) and 64 GB DDR4, running Ubuntu20.04. Used as reference point and for comparison versus the benchmarked edge solutions used in this work.

2. RP4_64bit (Raspberry Pi 4 Model B 8 GB) [52]

The main compute element of Raspberry Pi 4 Model B was its quad-core ARM Cortex-A72 CPU that supports NEON 128-bit wide vector instructions, running at a clock speed of 1.5 GHz. This variant (RP4_64bit) was fitted with 8 GB LPDDR4 and was running a 64bit OS (Bullseye).

3. RP4_32bit (Raspberry Pi 4 Model B 4 GB) [52]

Similar to RP4_64bit, but with the main difference of having 4 GB LPDDR4 and running a 32-bit OS (Buster).

4. Nano (NVIDIA Jetson Nano) [53]

NVIDIA Jetson Nano (Nano) includes an embedded GPU with 128 CUDA cores, a quad-core ARM Cortex-A57 64-bit CPU, and 4 GB LPDDR4. From the two power modes supported, we used the power mode MAXN (10 Watts) where the 4× CPU cores ran at 1.48 GHz and the GPU at 921.6 MHz.

5. XavierNX (NVIDIA Jetson Xavier NX) [54]

NVIDIA Jetson Xavier NX (XavierNX) is a more powerful family compared to Nano, as it includes more GPU cores, a more powerful CPU, and higher density and speed LPDDR4. In particular, its GPU includes 384-cores and 48 Tensor Cores, while its CPU is a 64-bit 6-core NVIDIA Carmel ARMv8.2. From the various power modes it supports, we used the power mode 6 (XavierNX:6; 20 Watts, 2× cores at 1.9 GHz/GPU at 1.1 GHz) and power mode 8 (XavierNX:8; 20 Watts, 6× cores at 1.4 GHz/GPU at 1.1 GHz).

## 5. Experimental Results

The experimental results section is divided into several subsections. Section 5.1 explains the evaluation metrics used, followed by the benchmarking results for each of the hardware platforms using EVM and rPPG approaches. Section 5.2 and Section 5.3 present the latency figures of each stage depicted in Figure 1. Section 5.4 shows the various power consumption measurements during idle and runtime operation. Finally, Section 5.5 compares total throughput, value (throughput/cost), and efficiency (throughput/Watt) of each hardware platform. Together, these subsections provide a comprehensive analysis of the performance of each vital sign estimation method on different hardware platforms.

### 5.1. Evaluation Metrics

The metrics used to evaluate the performance of each hardware device is described below:

- Latency: The time to execute a stage from start to finish measured in milliseconds (ms). To accurately extract the execution time, each stage was performed multiple times and the average time was logged. Apart from this software process, other OS processes use the hardware resources too (such as CPU cores, cache memory, etc.) and they can add potential noise to our results if not run a sufficient number of times.
- Throughput: Total amount of frames per second (FPS) that can be processed every second. The FPS metric is calculated via Equation (3), where Total Latency is the total execution time including all stages from start to finish for each approach.

(3) $$FPS=\frac{Time}{TotalLatency}=\frac{1}{TotalLatency}$$

- Power Consumption: Power consumption (Watts) was measured with a power meter. Average power consumption was recorded for the idle state and additionally for each of the three resolutions.
- Value: Throughput/cost is calculated with Equation (4), where FPS is the number of processed frames per second as explained previously and cost is the financial price of the hardware board in US dollars.

(4) $$Value=\frac{FPS}{Cost}$$

- Efficiency: Throughput/Watt is calculated with Equation (5), where FPS is the number of processed frames per second as explained previously and Average Power is the mean power consumption reading of the three video resolutions.

(5) $$Efficiency=\frac{FPS}{AveragePower}$$

### 5.2. EVM Latency Results

Detailed hardware latency results obtained for the EVM approach with BlazeFace and FaceMesh are presented in Table 3 and Table 4, respectively. Regarding the CNN model latency performance, FaceMesh was on average x1.8 more compute demanding, which in turn resulted in an average ×0.8 less total throughput (FPS) in comparison to BlazeFace. Additionally, the ‘Post-Process’ stage was ×3.9 times slower with the later model because the cropping and masking of the ROIs from the original frame was more complex (forehead, left/right cheek). The rest of the stages had relatively close latency figures between each other.

**Figure 5:**
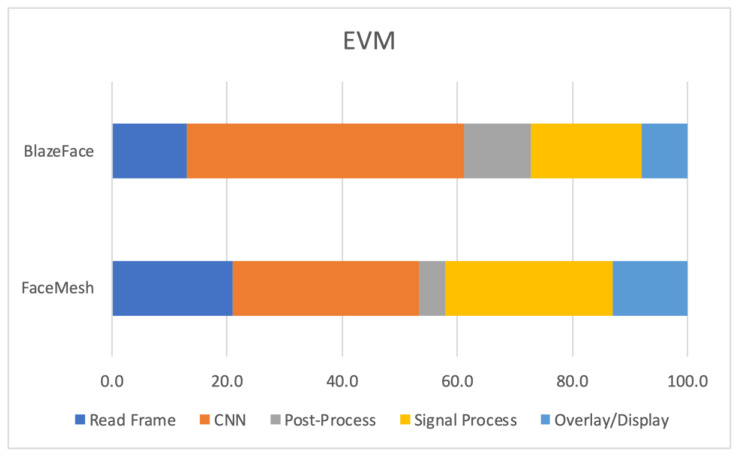
Average results of all edge platforms showcasing percentage of the total time spent on executing each stage of EVM with BlazeFace and FaceMesh.

An examination of the duration of execution for each stage (averaged out across all edge devices) in relation to the overall processing time can provide valuable insights into identifying the bottlenecks or stages which contribute to the majority of the processing time. The findings, presented in Figure 5, indicate that the BlazeFace implementation expended an average of 20.9% of the total processing time on ‘Read Frame’, followed by 32.5% on ‘CNN’, 4.5% on ‘Post-Process’, 22.1% on ‘Signal Process’, and 13.0% on ‘Overlay/Display’. It is noteworthy that the ‘CNN’ stage accounted for almost one third of the total processing time, whereas the second most computationally intensive stage was the ‘Post-Process’.

In contrast, the FaceMesh implementation consumed an average of 13.0% of the total processing time on ‘Read Frame’, followed by 48.1% on ‘CNN’, 11.7% on ‘Post-Process’, 19.1% on ‘Signal Process’, and 7.9% on ‘Overlay/Display’. The results demonstrate a difference in the processing time distribution between the two implementations, with the ‘CNN’ stage being the most computationally demanding stage in the FaceMesh implementation, followed by the ‘Post-Process’ stage.

Below, we provide some insightful observations when both models run on the edge devices. Firstly, as the resolution is decreased, the ‘CNN’ stage is also decreased, but the ‘Post-Process’ time is increased. Regarding the ‘CNN’ stage, this can be explained from the fact that, as the video resolution decreased, so did the computational toll of resizing the image, as this was a two-step stage. Regarding the ‘Post-Process’, when ROIs were extracted from the 640 × 360 resolution, this resulted in requiring upscaling due to very small bounding boxes, which in turn increased the latency times of this stage. Secondly, the FPS did increase as the resolution decreased, specifically on average a 20% increase was seen from 1920 × 1080 to 1280 × 720, and 38% with 1920 × 1080 to 640 × 360.

In terms of the fastest and slowest edge platforms for BlazeFace + EVM, XavierNX:8 had an average of 39.3 FPS, while RP4_32bit had only 11.7 FPS. As for FaceMesh + EVM, the fastest edge platform was XavierNX:6 with 36.4 FPS, while the slowest one was RP4_32bit with 7.6 FPS.

### 5.3. rPPG Latency Results

Table 5 and Table 6 present the edge hardware results obtained for rPPG approach with BlazeFace and FaceMesh, respectively. FaceMesh was on average ×1.8 more compute demanding, which in turn resulted on average ×0.5 less total throughput (FPS) in comparison to BlazeFace. Additionally, the ‘Post-Process’ stage was ×3.4 times slower with the later model, while the rest of the stages were relatively close to each other.

**Figure 6:**
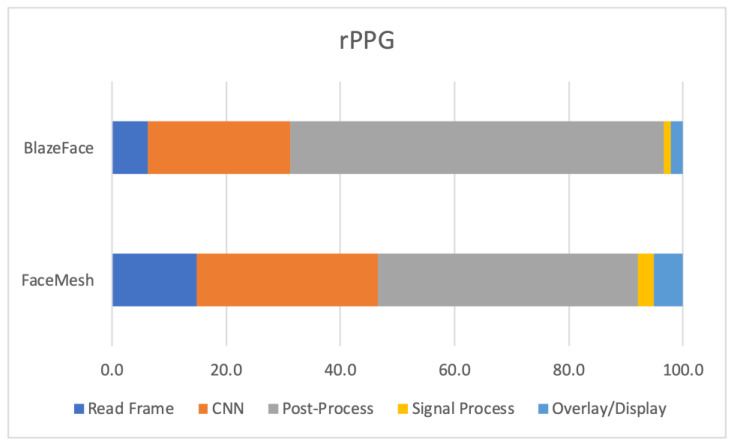
Average results of all edge platforms showcasing percentage of the total time spent on executing each stage of rPPG with BlazeFace and FaceMesh.

Figure 6 presents the percentage of total execution time of each stage (averaged out across all edge devices) for the rPPG approach, where bottlenecks can be identified. For the BlazeFace implementation, an average of 14.9% of the total processing time was allocated to the ‘Read Frame’ stage, followed by 31.7% on ‘CNN’, 45.5% on ‘Post-Process’, 2.7% on ‘Signal Process’, and 5.2% on ‘Overlay/Display’. Notably, the ‘Post-Process’ stage was the most computationally demanding stage (23.4% more compared to EVM), while the ‘CNN’ stage was the second most resource intensive (13% more compared to EVM).

Moreover, for the FaceMesh implementation, an average of 6.3% of the total processing time was devoted to the ‘Read Frame’ stage, followed by 25.0% on ‘CNN’, 65.5% on ‘Post-Process’, 1.1% on ‘Signal Process’, and 2.2% on ‘Overlay/Display’. It is noteworthy that the ‘Post-Process’ stage was the most computationally intensive stage in this scenario, followed by the ‘CNN’ stage.

Similar to what was observed with EVM results, as the resolution is decreased, the ‘CNN’ latency also decreased, but the ‘Post-Process’ time increased. For BlazeFace + rPPG, the fastest edge platform was XavierNX:6 with 34.6 FPS, while the slowest one was RP4_32bit with 11.3 FPS. As for FaceMesh + rPPG, the fastest edge platform was XavierNX:6 with 19.5 FPS, while the slowest one was RP4_32bit with 5.2 FPS.

### 5.4. Power Consumption Results

Table 7 presents the meter readings of power consumption across different platforms, encompassing the idle state (i.e., no active processes), three video resolutions, and an average power consumption value. The results indicate that, with the exception of PC, the platform with the lowest power consumption on average was RP4_32bit, registering 4.9 Watts, while the platform with the highest power consumption was XavierNX:8, with an average of 10.0 Watts. In general, a 4.3% drop in power was observed when downscaling to 1280 × 720 and 6% drop when downscaling to 640 × 360 from 1920 × 1080 resolution.

### 5.5. FPS, Efficiency, and Value Results

Table 8 provides an alternative perspective on the capabilities of each edge platform, taking into account their cost and power consumption in relation to their throughput. Specifically, the focus of the analysis is on the efficiency metric (throughput/Watt) and the value metric (throughput/cost), which are calculated based on the average FPS, average power consumption, and cost of each device. For both EVM and rPPG, XavierNX:6 platform came out on top on efficiency and RP4_64bit in terms of value in every case.

## 6. Conclusions

In this paper, we have evaluated the performance of four off-the-shelf edge platforms by implementing two algorithmic approaches for estimating the heart rate and respiratory rate of an individual. Compared to traditional methods, we have used two contactless methods, by utilizing RGB cameras and AI in order to detect ROIs of an individual’s face. The results showcase the capabilities of various edge hardware platforms by using several metrics, the baseline performance people should expect when using Eulerian Video Magnification and Remote Photoplethysmography in real-time edge applications, as well as the performance of different application steps.

These findings contribute to the field of AI-based health monitoring and have practical implications for implementing systems that are able to estimate vital signs of patients without any contact, in order to lower financial costs, reduce visit times, increase comfort, and enhance safety for healthcare professionals.

Regarding the hardware performance for both the EVM and rPPG method, the XavierNX platform outperformed all other evaluated embedded boards in terms of latency, throughput, and efficiency because of its advantageous CPU and accelerator; meanwhile, in terms of value and power consumption, the RP4_64bit was found to outperform the other tested boards. Moreover, the most computationally expensive part of the pipeline for EVM was found to be the ‘CNN’, while for rPPG it was the ‘Post-Process’ stage.

However, there are still several challenges and limitations associated with the use of EVM and rPPG methods on edge hardware. While platforms such as NVIDIA Jetson Nano, Xavier NX, and RP4_64bit are able to achieve 30 FPS and more, they must scale down the video resolution which could affect the quality of the image and hence introduce noise to the results. Additionally, there are various improvements that can be implemented in order to increase throughput, by optimizing bottlenecks using hardware specific resources (hardware optimized models, parallelization, threading, dimension reduction techniques, etc.), but there are limitations in how accurate the algorithms can be. This opens avenues for future research that could be built on this study, such as exploring 2D or 3D CNNs to estimate vital signs from RGB video streams in terms of both accuracy and edge hardware performance. A lot of the data pre-processing and algorithms could be replaced by an AI model that could assist in reducing computational complexity, making it more suitable for resource-constrained devices.

Overall, this study has made a valuable contribution to the field of AI-based health monitoring and provides a starting point for further research and development in this area. We hope that these findings will inform and guide the development of heart rate and respiratory rate estimations via contactless methods, leading to more advanced and effective solutions in the future.

## Figures and Tables

**Table 1:** Summary of rPPG signal extraction methods [21].

rPPG Method	Summary
GREEN [22]	Of the three channels, the green channel is most likely the PPG signal and can be used as its estimate.
ICA [23]	To recover three separate source signals, independent component analysis (ICA) is applied to the RGB signal. A significant rPPG signal was usually found in the second component.
PCA [24]	Principal component analysis (PCA) is applied to distinguish the rPPG signal from the RGB signal.
CHROM [25]	The chrominance (CHROM)-based method generates an rPPG signal by removing the noise caused by the light reflection using a ratio of the normalized colour channels.
PBV [26]	PBV calculates the rPPG signal with blood-volume pulse fluctuations in the RGB signal to identify the pulse-induced colour changes from motion
POS [27]	The plane-orthogonal-to-skin (POS) method uses the plane orthogonal to the skin tone in the RGB signal to extract the rPPG signal.
LGI [28]	The local group invariance (LGI) calculates an rPPG signal with a robust algorithm as a result of local transformations.
OMIT [29]	Orthogonal matrix image transformation (OMIT) recovers the rPPG signal by generating an orthogonal matrix with linearly uncorrelated components representing the orthonormal components in the RGB signal, relying on matrix decomposition.

**Table 2:** List of benchmarked hardware platforms and their core specifications.

#	Hardware	CPU	Cores/Frequency	Memory	AI Accelerator
1	PC	Intel i9-9900K	8/3.6 GHz	64 GB (LPDDR4)	GPU: GTX1060
2	RP4_32bit	ARM Cortex-A72	4/1.5 GHz	4 GB (LPDDR4)	N/A
3	RP4_64bit	ARM Cortex-A72	4/1.5 GHz	8 GB (LPDDR4)	N/A
5	Nano	ARM Cortex-A57	4/1.48 GHz	4 GB (LPDDR4)	GPU: 128-core Maxwell
6	XavierNX	Carmel ARM^®^v8.2	6/1.42–1.9 GHz	8 GB (LPDDR4)	GPU: 384-core Volta

**Table 3:** Latency results of EVM with BlazeFace on each hardware platform.

Hardware	Resolution	Read Frame (ms)	CNN (ms)	Post-Process (ms)	Signal Process (ms)	Overlay/Display (ms)	FPS	Average FPS
PC	1920 × 1080	1.66	2.63	0.16	2.61	0.36	134.18	156.5
	1280 × 720	0.76	2.38	0.14	2.63	0.36	158.96	
	640 × 360	0.17	2.22	0.26	2.64	0.36	176.4	
RP4_32bit	1920 × 1080	29.66	26.6	3.30	21.45	9.34	11.05	11.7
	1280 × 720	13.67	24.22	3.30	29.17	17.19	11.41	
	640 × 360	4.19	24.93	3.45	29.08	17.24	12.66	
RP4_64bit	1920 × 1080	15.55	20.25	1.34	13.41	5.08	17.94	22.3
	1280 × 720	8.21	16.15	1.33	13.28	5.16	22.64	
	640 × 360	2.41	14.94	2.16	13.50	5.12	26.20	
Nano	1920 × 1080	13.7	15.37	1.40	14.19	5.64	19.67	24.0
	1280 × 720	6.89	12.41	1.42	14.48	5.63	24.28	
	640 × 360	1.91	11.13	2.02	14.44	5.58	28.18	
XavierNX:6	1920 × 1080	15.89	12.26	1.41	5.31	2.00	26.43	39.2
	1280 × 720	8.27	8.86	1.28	5.71	2.18	37.34	
	640 × 360	1.47	6.92	2.96	4.61	2.04	53.91	
XavierNX:8	1920 × 1080	13.35	9.68	1.06	5.54	2.43	30.60	39.3
	1280 × 720	6.83	7.68	1.04	5.77	2.59	40.81	
	640 × 360	1.44	7.98	3.32	5.59	2.52	46.57	

**Table 4:** Latency results of EVM with FaceMesh on each hardware platform.

Hardware	Resolution	Read Frame (ms)	CNN (ms)	Post-Process (ms)	Signal Process (ms)	Overlay/Display (ms)	FPS	Average FPS
PC	1920 × 1080	1.56	3.51	1.08	2.78	0.35	107.24	121.8
	1280 × 720	0.68	3.26	1.00	2.78	0.35	123.19	
	640 × 360	0.19	3.02	1.02	2.79	0.36	134.90	
RP4_32bit	1920 × 1080	30.66	63.98	12.65	21.67	7.93	7.29	7.6
	1280 × 720	14.55	61.37	10.84	29.38	16.55	7.52	
	640 × 360	4.34	61.58	12.75	29.37	16.52	8.01	
RP4_64bit	1920 × 1080	15.30	28.91	6.15	13.05	4.59	14.68	17.2
	1280 × 720	8.12	25.55	6.19	13.11	4.72	17.30	
	640 × 360	2.47	24.36	6.46	13.20	4.64	19.52	
Nano	1920 × 1080	13.29	54.27	10.82	13.99	4.37	10.26	11.4
	1280 × 720	6.32	50.12	10.75	14.07	4.36	11.59	
	640 × 360	1.92	49.68	10.87	14.00	4.40	12.26	
XavierNX:6	1920 × 1080	11.16	12.76	4.67	4.71	1.86	27.62	36.4
	1280 × 720	4.59	10.55	4.45	4.79	2.00	37.05	
	640 × 360	1.38	9.66	4.82	4.06	1.95	44.48	
XavierNX:8	1920 × 1080	10.45	12.05	5.28	5.19	2.33	27.76	32.7
	1280 × 720	5.39	11.48	5.09	5.17	2.40	33.08	
	640 × 360	1.70	10.98	5.79	5.30	2.43	37.19	

**Table 5:** Latency results of rPPG with BlazeFace on each hardware platform.

Hardware	Resolution	Read Frame (ms)	CNN (ms)	Post-Process (ms)	Signal Process (ms)	Overlay/Display (ms)	FPS	Average FPS
PC	1920 × 1080	1.69	3.78	11.44	0.33	0.82	49.37	66.1
	1280 × 720	0.94	3.69	7.49	0.41	0.46	71.98	
	640 × 360	0.53	7.28	4.40	0.85	0.38	77.03	
RP4_32bit	1920 × 1080	37.04	62.62	86.02	1.58	8.50	3.95	11.3
	1280 × 720	16.50	36.50	36.40	1.45	4.10	8.32	
	640 × 360	4.51	22.86	9.40	1.54	2.84	21.71	
RP4_64bit	1920 × 1080	18.75	24.33	54.92	1.50	5.21	8.17	19.3
	1280 × 720	8.62	17.90	25.25	1.40	3.08	15.41	
	640 × 360	2.38	14.00	6.61	1.45	2.77	34.44	
Nano	1920 × 1080	14.54	24.48	64.05	1.87	2.84	8.42	22.2
	1280 × 720	6.48	15.78	29.30	1.74	2.06	16.61	
	640 × 360	1.91	10.44	7.85	1.70	1.63	41.46	
XavierNX:6	1920 × 1080	10.94	16.09	33.66	2.20	3.18	13.71	34.6
	1280 × 720	4.94	12.19	14.12	1.65	2.56	26.12	
	640 × 360	1.39	7.95	3.72	1.65	2.20	63.87	
XavierNX:8	1920 × 1080	10.26	14.97	37.14	1.96	3.23	13.20	31.0
	1280 × 720	5.00	11.44	16.32	1.93	2.91	24.69	
	640 × 360	1.42	8.37	4.47	1.90	3.00	55.18	

**Table 6:** Latency results of rPPG with FaceMesh on each hardware platform.

Hardware	Resolution	Read Frame (ms)	CNN (ms)	Post-Process (ms)	Signal Process (ms)	Overlay/Display (ms)	FPS	Average FPS
PC	1920 × 1080	1.70	4.40	38.04	0.31	0.91	22.11	41.7
	1280 × 720	0.86	3.99	18.27	0.31	0.53	43.17	
	640 × 360	0.37	6.57	10.68	0.55	0.34	59.76	
RP4_32bit	1920 × 1080	37.78	100.59	316.65	1.46	8.49	2.15	5.2
	1280 × 720	16.93	74.52	145.67	1.48	4.17	4.13	
	640 × 360	4.74	60.64	39.57	1.53	2.69	9.36	
RP4_64bit	1920 × 1080	18.64	33.58	169.92	1.39	5.00	4.38	10.6
	1280 × 720	8.71	27.75	78.04	1.44	3.34	8.43	
	640 × 360	2.60	23.74	22.78	1.43	2.64	19.04	
Nano	1920 × 1080	14.29	62.85	186.31	1.73	2.78	3.79	7.9
	1280 × 720	6.43	54.31	88.08	1.74	2.06	6.76	
	640 × 360	1.91	49.15	28.14	1.73	1.63	13.04	
XavierNX:6	1920 × 1080	10.13	17.90	103.48	1.69	2.85	7.51	19.5
	1280 × 720	4.50	13.18	49.42	1.59	2.52	14.54	
	640 × 360	1.39	9.86	15.04	1.56	2.24	36.45	
XavierNX:8	1920 × 1080	9.60	16.97	136.13	1.96	3.71	6.04	15.8
	1280 × 720	4.59	13.24	65.53	1.88	3.17	11.67	
	640 × 360	1.40	10.78	19.71	1.85	3.05	29.61	

**Table 7:** Power consumption hardware platform results.

Hardware	Idle (Watt)	1920 × 1080 (Watt)	1280 × 720 (Watt)	640 × 360 (Watt)	Average (Watt)
PC	49.4	120	111	104	111.7
RP4_32bit	3.4	5.2	4.9	4.6	4.9
RP4_64bit	3.4	5.8	5.5	5.2	5.5
Nano	3.8	6.5	5.9	5.7	6.0
XavierNX:6	6.7	9.5	9.1	9.1	9.2
XavierNX:8	6.9	10.0	9.9	10.0	10.0

**Table 8:** Average FPS, efficiency, and value hardware platform results.

Hardware	EVM						rPPG					
	BlazeFace			FaceMesh			BlazeFace			FaceMesh		
	FPS	Efficiency	Value	FPS	Efficiency	Value	FPS	Efficiency	Value	FPS	Efficiency	Value
PC	156.5	1.40	0.08	121.8	1.09	0.06	66.1	0.59	0.03	41.7	0.37	0.02
RP4_32bit	11.7	2.39	0.21	7.6	1.55	0.14	11.3	2.31	0.21	5.2	1.06	0.09
RP4_64bit	22.3	4.05	**0.30**	17.2	3.12	**0.23**	19.3	3.52	**0.26**	10.6	1.93	**0.14**
Nano	24.0	3.99	0.16	11.4	1.88	0.08	22.2	3.67	0.15	7.9	1.30	0.05
XavierNX:6	39.2	**4.25**	0.10	**36.4**	**3.94**	0.09	**34.6**	**3.74**	0.09	**19.5**	**2.11**	0.05
XavierNX:8	**39.3**	3.95	0.10	32.7	3.28	0.08	31.0	3.11	0.08	15.8	1.58	0.04

## Data Availability

Not applicable.
